# Barcoding the Caatinga biome bees: a practical review

**DOI:** 10.1007/s11033-025-10307-7

**Published:** 2025-02-04

**Authors:** Pedro Rodrigues, Cláudia Teixeira, Laura Guimarães, Nuno G. C. Ferreira

**Affiliations:** 1 CIIMAR - Interdisciplinar Centre of Marine and Environmental Research, University of Porto, Terminal de Cruzeiros do Porto de Leixões, Av. General Norton de Matos, Matosinhos, 4450-208 Portugal; 2 Cardiff University– School of Biosciences, Museum Avenue, Cardiff, CF10 3AX, Wales UK

**Keywords:** Caatinga biome, Hymenoptera, Native bees, Barcoding

## Abstract

Bees play a critical role as pollinators in ecosystem services, contributing significantly to the sexual reproduction and diversity of plants. The Caatinga biome in Brazil, home to around 200 bee species, provides an ideal habitat for these species due to its unique climate conditions. However, this biome faces threats from anthropogenic processes, making it urgent to characterise the local bee populations efficiently. Traditional taxonomic surveys for bee identification are complex due to the lack of suitable keys and expertise required. As a result, molecular barcoding has emerged as a valuable tool, using genome regions to compare and identify bee species. However, little is known about Caatinga bees to develop these molecular tools further. This study addresses this gap, providing an updated list of 262 Caatinga bee species across 86 genera and identifying ~ 40 primer sets to aid in barcoding these species. The findings highlight the ongoing work needed to fully characterise the Caatinga biome’s bee distribution and species or subspecies to support more effective monitoring and conservation efforts.

**Supplementary Information:**

The online version contains supplementary material available at 10.1007/s11033-025-10307-7.

## Introduction

Pollinators play a crucial role in ecosystem health, contributing to plant diversity, agricultural production, and overall vitality [1]. Insects, especially bees, are the primary pollinators [1, 2], with over 20,000 bee species worldwide [3]. However, bee populations are declining, largely due to human activities, with potentially catastrophic consequences for ecosystems. Given the richness of its ecosystems and Brazil’s location, this country holds around one-fourth of the total bee species [4]. The country is also a major honey producer thanks to its floral diversity and climate [5]. The Caatinga biome, in particular, is characterised by abundant nectar and pollen sources that sustain bee populations year-round [6]. Despite the importance of bees, information on their abundance and diversity in Brazil is limited. What is known is that bee populations have been declining in the region [7, 8]. Native stingless bees (Meliponini) are responsible for pollinating 90% of Brazil’s native trees [9], underscoring the critical need to conserve these essential pollinators.

Bee biodiversity and abundance are important indicators of ecological integrity and environmental health, signalling the need for management and protection. However, accurately identifying bee species is crucial for assessing these indicators and investigating species ecology, tolerance, and evolution [10]. Unfortunately, bee identification is challenging due to limited taxonomic resources and expertise. DNA barcoding offers a valuable alternative, using a specific segment of an organism’s DNA as a unique identifier [10]. This DNA “fingerprint” can be compared to databases of known species, enabling rapid and accurate species identification [10, 11]. DNA barcoding has numerous practical applications, from disease and pest control to food traceability, conservation, and resource management. Several databases have been created to store DNA barcode sequences, which researchers have used to identify bees and even discover new species. Additionally, the non-invasive sampling enabled by DNA barcoding can facilitate broader taxonomic investigations. However, the existing primer sets for bee identification remain dispersed and in need of systematisation [12].

This review aims to compile the most suitable PCR primer sets for DNA barcoding of native and non-native bee species in the Caatinga biome. By gathering this information, the study seeks to promote and streamline the integration of bee DNA barcoding into routine monitoring and research evaluations. Given the importance of improving knowledge, monitoring, and assessment of Caatinga bees, this work also provides complete characterisations of the biome and endemic Brazilian Caatinga alongside the primer details.

## Methodology

A comprehensive literature search was conducted using the SCOPUS and Portal de Periódicos da CAPES (PC) databases to identify papers published between 1991 and 2024. The search followed the Preferred Reporting Items for Systematic Reviews and Meta-Analyses (PRISMA) guidelines, querying titles, abstracts, and keywords for the terms “Caatinga” and “bees.” This yielded 159 records from Scopus and 260 from CP. Two additional records were included as they were cited in other manuscripts (see Figure SD1 in Supplementary Material). Retracted and editorial documents were excluded. After removing duplicates, 279 records were screened based on the following criteria: (1) specific location within the Caatinga region, (2) inclusion of bee sampling and identification methodology, and (3) identification of bees to at least the species level. Records were excluded if the study was conducted in a transition area between the Caatinga and other biomes or if they only reported kleptoparasitic bee species. This screening process resulted in 161 records being removed, leaving 85 peer-reviewed publications [6, 13–97] in the final dataset. A complete list of the included publications and the bee species described in them is provided in Supplementary Material (Table SD1).

A comprehensive review of primer combinations effectively used for DNA barcoding across different genera was conducted using data from the BOLD database [98]. This list was supplemented with data from other studies, identified through a search through the Web of Science engine, focusing on species described within the Caatinga biome.

## The Caatinga biome

**Fig. 1:**
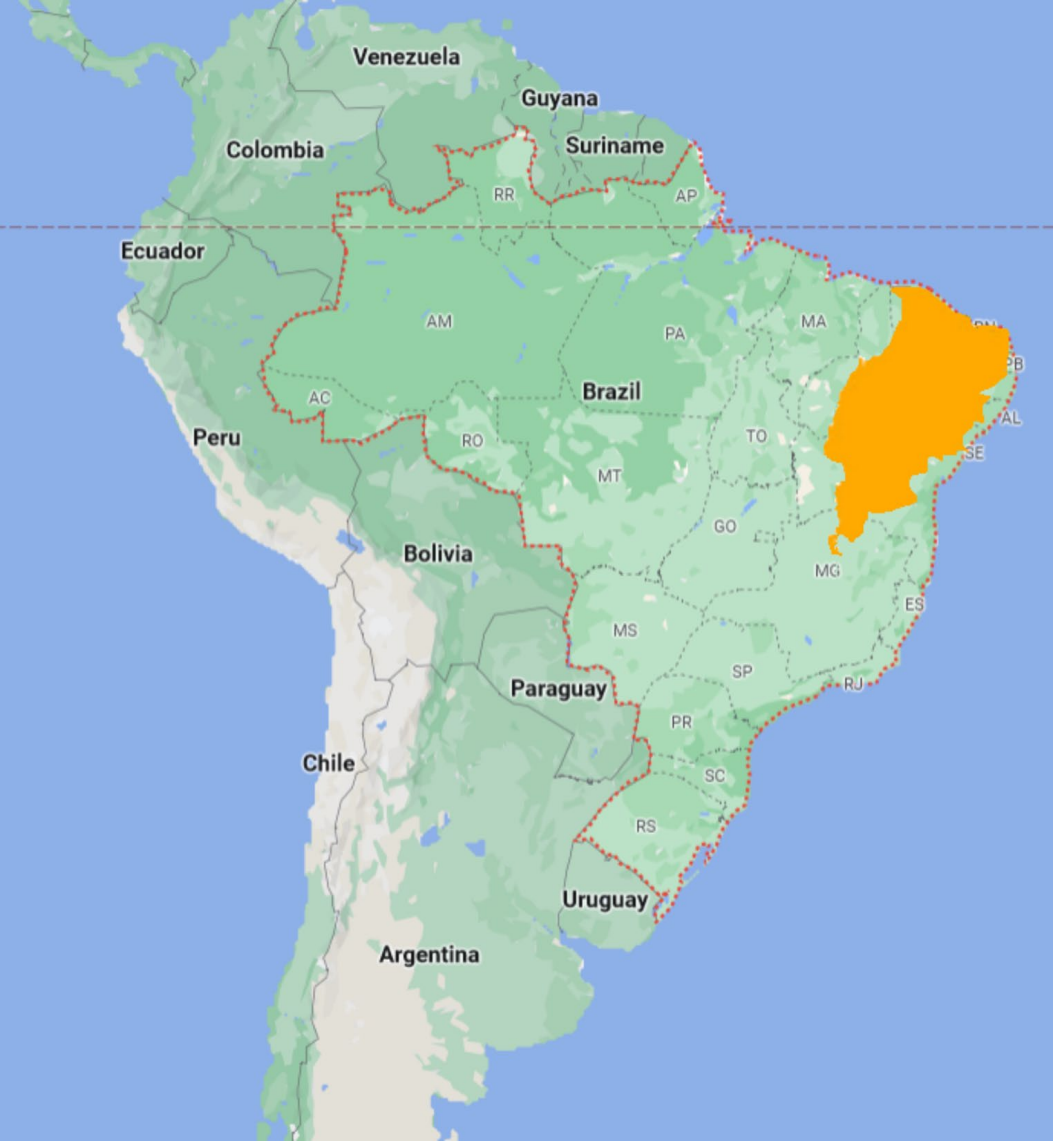
Map of the Caatinga biome. The area is restricted to northeastern Brazil. (Image adapted from Google Earth^®^)

The Caatinga biome is an unique biome found in northeastern Brazil, covering nearly 800,000 km^2^ [20] and encompassing 12% of the country’s territory (Fig. 1) [99]. This semi-arid region is characterised by leaf-shedding vegetation and the remarkable regenerative capacity of its species [99]. Spanning nine Brazilian states Piauí, Ceará, Rio Grande do Norte, Paraíba, Pernambuco, Alagoas, Sergipe, Bahia, and Minas Gerais [100], the Caatinga is known for its complex and extreme climate [4, 101], with high solar radiation, temperatures, and low, irregular rainfall [5] often experiencing 7–9 dry months per year [102]. Despite being home to over 20 million people, the Caatinga is the Brazilian biome most impacted by anthropogenic activities [103], jeopardising its rich biodiversity [23, 104]. The biome supports a high diversity of nectar and pollen-rich plants that flower year-round, providing a continuous food source for bees [105]. However, the prolonged dry season also leads to a scarcity of essential pollen types, impacting bee populations. Although the Caatinga is of great economic and environmental significance, relatively little is known about its native bee species compared to other Brazilian biomes.

## Main bee species found in the Caatinga biome

Brazil boasts an impressive diversity of bee species, with approximately a quarter of the world’s bee species found within its borders. This is due to the country’s vast array of ecosystems and favourable geographic location. Brazil’s rich floral diversity and climate provide immense potential for further study of its bee populations. Notably, around 70% of all bees are solitary species that do not live in hives or produce honey [106]. However, these solitary bees are critical in pollinating both agricultural and natural systems [107]. Bees rely on nectar and pollen to support their life cycles, which are closely synchronised with the flowering patterns of their host plants [106].

**Fig. 2:**
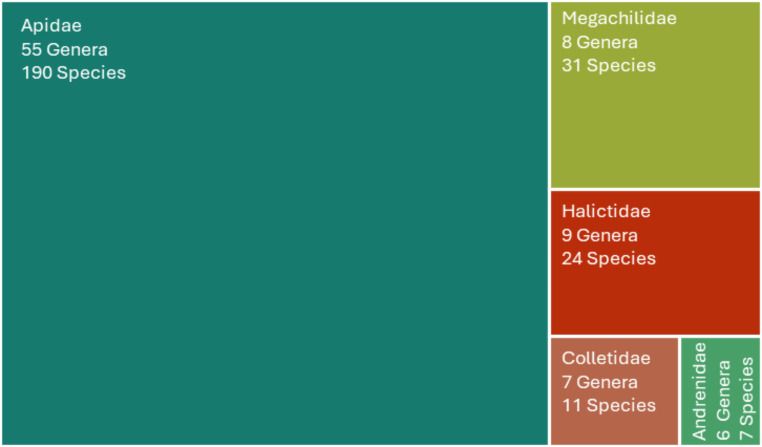
Updated treemap showing the Families and the corresponding number of genera and species identified for the Caatinga biome

This study provides a new comprehensive review identifying 262 non-parasitic bee species across 86 genera within the Caatinga biome (see Supplementary Data for the complete list). This marks a substantial increase from the 187 species and 77 genera previously reported by Zanella et al. [6]. The preliminary overview in Fig. 2 showcases this new data on recorded bee species in the Caatinga region. The review found that five out of the seven bee families were recorded, including Megachilidae (31 species, 8 genera), Halictidae (24 species, 9 genera), Colletidae (11 species, 7 genera), Apidae (189 species, 55 genera), and Andrenidae (7 species, 6 genera). While the number of new species added has increased by 40%, the species-per-genus ratio remains low for some families (1.17 Andrenidae < 1.57 Colletidaee < 2.67 Halictidae < 3.43 Apidae < 3.88 Megachilidae) as also previously reported by Zanella et al. [6]. This is particularly evident in the Andrenidae family, where only one species per genus was recorded, except for *Acamptopoeum*, which has two species. In contrast, two-thirds of the genera within the Megachilidae family have two or more species recorded. The updated species list is based on collected or observed specimens, which may not cover the entire bee fauna in the Caatinga biome. Additionally, many publications have only identified these organisms up to the genus level, highlighting the need for further studies using DNA barcoding techniques to improve species-level identification.

## Primers and barcode of Caatinga bee species

DNA barcoding is a valuable tool that allows the identification of specific species through DNA analysis [10]. This technique can be used with traditional taxonomic identification, providing additional information for further research, such as phylogenetic studies. However, barcoding heavily relies on properly curated databases to compare the data [10]. Barcoding can be particularly useful when traditional taxonomy is challenging, such as when organisms are incomplete and lack the specific characteristics needed for identification or when sexual dimorphism or life cycle changes confound the process. Additionally, barcoding can aid in identifying new or unknown species and cataloguing taxa in a specific area, helping to determine species boundaries [10, 108, 109]. The updated list of bee species indicates that the Caatinga biome shelters many more bee species than previously thought. DNA barcoding can help provide a more realistic and comprehensive understanding of the biodiversity in this region.

The cytochrome C Oxidase Subunit I is a widely used barcode region for Hymenoptera, the insect order that includes bees [110]. While more than 65 primers have been identified in the literature, only 61 have been successfully confirmed, as shown in Table 1. These primers have been used in 22 studies [111–132], with various combinations of forward and reverse primers, or primer cocktails, employed to perform successful amplifications. In some cases, primers specific to other taxa, such as Lepidoptera, have also been utilised for barcoding [115]. Table 2 presents the list of primer combinations reported to successfully barcode species of different genera based on data available on the BOLD database [98].

**Table 1:** Primer sequence and corresponding references used for the barcode of bee species inhabiting the Caatinga biome

Primer name	Primer sequence(5’-3’)	Reference		Primer name	Primer sequence(5’-3’)	Reference
AMR1deg_R	CAWCCWGTWCCKRMNCCWKCAT	[31]		LCO1490_t1	TGTAAAACGACGGCCAGTGGTCAACAAATCATAAAGATATTGG	[31]
AncientLepF2	ATTGGWGATGATCAAATTTATAAT	[40]		LCO1490-JJ	CHACWAAYCATAAAGATATYGG	[48]
BarbeeF	CAACAAATCATAAAAATATTGG	[46]		LCOLong	TCAACAAATCATAAAGATATTGGWAT	[41]
BEEf	TWYTCWACWAAYCATAAAGATATTGG	[37]		LepF1	ATTCAACCAATCATAAAGATATTGG	[28]
BEEr	TGATTTTTTGGWCAYCCWGAAGTWTA	[37]		LepF2_t1	TGTAAAACGACGGCCAGTAATCATAARGATATYGG	[44]
BterrestrisF	TATCAACTAATCATAAAAATATTGG	[49]		LepFoIF	RKTCAACMAATCATAAAGATATTGG	[40]
BterrestrisR	TATACTTCTGGGTGACCAAAAAATC	[49]		LepFoIR	TAAACTTCWGGRTGWCCAAAAAATCA	[40]
C_ANTMR1D	RonIIdeg_R: AMR1deg_R	[30]		LepR1	TAAACTTCTGGATGTCCAAAAAATCA	[28]
C_LepFolF	LepF1:LCO1490	[36]		LepRI_t1	CAGGAAACAGCTATGACTAAACTTCTGGATGTCCAAAAAATCA	[47]
C_LepFolR	LepR1:HCO2198	[36]		MHemF	GCATTYCCACGAATAAATAAYATAAG	[44]
C_microLepF1_t1	microLepF2_t1:microLepF3_t1	[40]		MHemR	GGTGGATAAACTGTTCAWCC	[44]
C_TypeR1	TypeR1:TypeR2:TypeR3	[40]		microLepF2_t1	TGTAAAACGACGGCCAGTCATGCWTTTATTATAATTTTYTTTATAG	[40]
C_VF1LFt1	VF1_t1:VF1d_t1:LepF1_t1:VF1i_t1	[47]		microLepF3_t1	TGTAAAACGACGGCCAGTCATGCWTTTGTAATAATTTTYTTTATAG	[40]
C_VR1LRt1	VR1_t1:VR1d_t1:LepR1_t1:VR1i_t1	[47]		microLepR2_t1	CAGGAAACAGCTATGACGTAATWGCWCCWGCTARWACWGG	[40]
C1-N-2191	CCCGGTAAAATTAAAATATAAACTTC	[38]		mlCOIintF	GGWACWGGWTGAACWGTWTAYCCYCC	[34]
Cer_COI_F	GSTTTATGAGCHGGAATANTAGG	[45]		MLepF1	GCTTTCCCACGAATAAATAATA	[32]
COI-C01	TYTCWACWAAYCAYAAAGAYATTGG	[39]		MLepF2_t1	TGTAAAACGACGGCCAGTGCWTTCCCMCGWATAAATAATATAAG	[40]
COI-C03	ACYTCYGGRTGACCAAARAAYCA	[39]		MLepR1	CCTGTTCCAGCTCCATTTTC	[32]
COI-HCO2198	AAACTTCAGGGTGACCAAAAAATCA	[35]		MLepR2	GTTCAWCCWGTWCCWGCYCCATTTTC	[40]
COI-LCO-1490	GTCAACAAATCATAAAGATATTGG	[35]		Nancy_short	CCCGGTAAAATTAAAATATAAAC	[43]
dgHCO-2198	TAAACTTCAGGGTGACCAAARAAYCA	[33]		RonIIdeg_R	GGRGGRTARAYAGTTCATCCWGTWCC	[31]
EnhLepR1	CTCCWCCAGCAGGATCAAAA	[32]		RonMWASPdeg_t1	TGTAAAACGACGGCCAGTGGWTCWCCWGATATAKCWTTTCC	[29]
HCO	TAAACTTCAGGGTGTCCAAAGAATCA	[38]		TypeR1	GGAGGRTAAACWGTTCAWCC	[40]
HCO2198	TAAACTTCAGGGTGACCAAAAAATCA	[35]		TypeR2	GGAGGGTAAACTGTTCAWCC	[40]
HCO2198_t1	CAGGAAACAGCTATGACTAAACTTCAGGGTGACCAAAAAATCA	[31]		VF1_t1	TGTAAAACGACGGCCAGTTCTCAACCAACCACAAAGACATTGG	[47]
HCO2198-JJ	AWACTTCVGGRTGVCCAAARAATCA	[48]		VF1d_t1	TGTAAAACGACGGCCAGTTCTCAACCAACCACAARGAYATYGG	[47]
HCO-Hymeno-2	TAWACTTCWGGRTGDCCAAAAAATCA	[42]		VF1i_t1	TGTAAAACGACGGCCAGTTCTCAACCAACCAIAAIGAIATIGG	[47]
Hymeno-1	TTTCWACAAATCWTAAAGATATTGG	[42]		VR1_t1	CAGGAAACAGCTATGACTAGACTTCTGGGTGGCCAAAGAATCA	[47]
LCO	TCAACAAATCATAAGGACATTGG	[38]		VR1d_t1	CAGGAAACAGCTATGACTAGACTTCTGGGTGGCCRAARAAYCA	[47]
LCO_Hym	TATCAACCAATCATAAAGATATTGG	[41]		VR1i_t1	CAGGAAACAGCTATGACTAGACTTCTGGGTGICCIAAIAAICA	[47]
LCO1490	GGTCAACAAATCATAAAGATATTGG	[35]				

**Table 2:** List of primer combinations previously used to successfully amplify organisms from each specific genus based on the BOLD Database System. The table is divided into family, subfamily, tribe, and genus/genera. Primers set grey cells represent genera where no data could be found

Family	Subfamily	Tribe	Genus/genera	Primers set
Andrenidae	Panurginae	Calliopsini	Acamptopoeum	LepF1/LepR1
Andrenidae	Panurginae	Calliopsini	Callonychium	LCO1490/HCO2198
Andrenidae	Oxaeinae		Oxaea	LepF1/LepR1
Andrenidae	Panurginae	Protandrenini	Psaenythia	LepF1/LepR1
Andrenidae	Panurginae	Protandrenini	Rhophitulus	LepF1/LepR1; C_LepFolF/C_LepFolR
Andrenidae	Panurginae	Protomeliturgini	Protomeliturga	
Apidae	Apinae	Apini	Apis	LepF1/LepR1; C_LepFolF/C_LepFolR; LCO1490/HCO2198; RonMWASPdeg_t1/LepR1; LepF1/C_ANTMR1D; LepFoIF/LepFoIR; LCO1490_t1/HCO2198_t1; LCO/HCO; MLepF1/C_LepFolR; LepFoIF/LepFoIR; C_LepFolF/MLepR2
Apidae	Apinae	Bombini	Bombus	LepF1/LepR1; RonMWASPdeg_t1/LepR1; LepF1/C_ANTMR1D; C_LepFolF/C_LepFolR; LCO1490-JJ/HCO2198-JJ; BEEf/BeeR; APOF2/C_Lep-Fol_R; BterrestrisF/BterrestrisR, MLepF1/C_LepFolR; C_LepFolF/MLepR2; LCO1490_t1/HCO2198_t1; Hymeno-1/Hymeno-2; C_microLepF1_t1/C_TypeR1; LCO_Hym/C1-N-2191; LepF1/EnhLepR1; LCO_Hym/Nancy_short; MLepF1/HCO2198_t1; MLepF2_t1/microLepR2_t1
Apidae	Apinae	Centridini	Centris	
Apidae	Apinae	Centridini	Epicharis	
Apidae	Apinae	Emphorini	Ancyloscelis	LepF1/LepR1; LepF1/EnhLepR1; C_LepFolF/C_LepFolR; RonMWASPdeg_t1/LepR1; LCO1490/HCO2198
Apidae	Apinae	Emphorini	Diadasia; Melioma; Melitomella; Ptilothrix	
Apidae	Apinae	Ericrocidini	Mesocheira; Mesoplia	
Apidae	Apinae	Eucerini	Melissodes	RonMWASPdeg_t1/LepR1; LepF1/C_ANTMR1D
Apidae	Apinae	Eucerini	Eucera; Florilegus; Gaesischia; Melissoptil; Melissoptila; Thygater; Trichocerapis	
Apidae	Apinae	Euglossini	Eufriesea; Euglossa; Eulaema; Exaerete	
Apidae	Apinae	Exomalopsini	Exomalopsis	
Apidae	Apinae	Isepeolini	Isepeolus	
Apidae	Apinae	Meliponini	Plebeia	LepF1/LepR1
Apidae	Apinae	Meliponini	Trigonisca	LepF1/LEPR1
Apidae	Apinae	Meliponini	Camargoia; Cephalotrigona; Frieseomelitta; Geotrigona; Lestrimelitta; Melipona; Paratrigona; Partamona; Plectoplebeia; Scaptotrigona; Tetragonisca; Trigona	
Apidae	Apinae	Osirini	Osirinus; Parepeolud	
Apidae	Apinae	Protepeolini	Leiopodus	
Apidae	Apinae	Rhathymini	Rhathymus	
Apidae	Apinae	Tapinotaspidini	Arhysoceble	LepF1/EnhLepR1
Apidae	Apinae	Tapinotaspidini	Caenonomada; Paratetrapedia; Tapinotaspoides	
Apidae	Apinae	Tetrapediini	Coelioxoides; Tetrapedia	
Apidae	Nomadinae	Brachynomadini	Brachynomada	
Apidae	Nomadinae	Epeolini	Rhogepeolus; Thalestria; Triepeolus	
Apidae	Xylocopinae	Ceratinini	Ceratina	
Apidae	Xylocopinae	Xylocopini	Xylocopa	C_LepFolF/C_LepFolR; LepF1/LepR1; LCO1490/HCO2198; LepF1/EnhLepR1; MLepF1/LepR1; LCO1490_t1/HCO2198_t1; RonMWASPdeg_t1/LepR1; LepF1/C_ANTMR1D; AncientLepF2/MLepR2; MLepF1/C_LepFolR; C_LepFolF/MLepR2; C_microLepF1_t1/C_TypeR1
Colletidae	Xeromelissinae		Chilicola	LepF1/LepR1; LepF1/C_ANTMR1D; RonMWASPdeg_t1/LepR1; LCO1490_t1/HCO2198_t1; MLepF1/LepR1
Colletidae	Colletinae		Colletes	dgHCO-2198/mlCOIintF; LepF1/LepR1; LepF1/C_ANTMR1D; RonMWASPdeg_t1/LepR1; LCO1490/HCO2198; LCO1490_t1/HCO2198_t1; C_LepFolF/C_LepFolR; BEEf/BEEr; LCOLong/C1-N-2191; LCO_Hym/C1-N-2191; MLepF1/LepR1; LepF1/MLepR1; COI-C01/COI-C03; MLepF1/C_LepFolR; C_LepFolF/MLepR2
Colletidae	Diphaglossinae	Dissoglottini	Mydrosomella	
Colletidae	Paracolletinae		Nomiocolletes	LepF1/LepR1
Colletidae	Paracolletinae		Perditomorpha; Protodiscelis; Sarocolletes	
Halictidae	Halictinae	Augochlorini	Augochlora	LepF1/LepR1; LCO1490/HCO2198; C_LepFolF/C_LepFolR; LepF1/MLepR1; MLepF1/LepR1; RonMWASPdeg_t1/LepR1; LepF1/C_ANTMR1D
Halictidae	Halictinae	Augochlorini	Augochlorella	LepF1/LepR1; C_LepFolF/C_LepFolR; LCO1490_t1/HCO2198_t1; LCO1490/HCO2198; MLepF1/C_LepFolR; MLepF1/LepR1; RonMWASPdeg_t1/LepR1; LepF1/C_ANTMR1D; C_LepFolF/MLepR2
Halictidae	Halictinae	Augochlorini	Augochloropsis	LepF1/LepR1; LCO1490/HCO2198; RonMWASPdeg_t1/LepR1; LepF1/C_ANTMR1D
Halictidae	Halictinae	Augochlorini	Pereirapis	
Halictidae	Halictinae	Augochlorini	Pseudaugochlora	LepF1/LepR1; LCO1490/HCO2198
Halictidae	Halictinae	Halictini	Halictus	LepF1/LepR1; AP0F1/APOR2; APOF2/APOR2; MLepF1/LepR1; LCO_Hym/Nancy_short; LCO1490/HCO2198; LCO1490_t1/HCO2198_t1; LCO_Hym/C1-N-2191; C_LepFolF/C_LepFolR; RonMWASPdeg_t1/LepR1; LepF1/C_ANTMR1D; LCO_Hym/Nancy_short; Hymeno2/Hymeno1; mlCOIintF/dgHCO-2198; LepF2_t1/MHemR; MHemF/LepR1; LCO1490_t1 /MLepR1
Halictidae	Halictinae	Halictini	Lasioglossum	LepF1/LepR1; BEEf/BEEr; RonMWASPdeg_t1/LepR1; LepF1/C_ANTMR1D; LCO1490_t1/HCO2198_t1; LCO1490/HCO2198; C_LepFolF/C_LepFolR; BarbeeF/C1-N-2191; Cer_COI_F/HCO2198_t1; LCO_Hym/Nancy_short; LCO_Hym/C1-N-2191; MLepF1/LepR1; MLepF1/C_LepFolR; C1-N-2191/BEEr; MLepF1/C_LepFolR; LepF1/MLepR1; C_LepFolF/MLepR2; MLepF1/HCO2198_t1; mlCOIintF /dgHCO-2198
Halictidae	Halictinae	Halictini	Agapostemon	LepF1/LepR1; LCO1490_t1/HCO2198_t1; LCO1490/HCO2198; LCO1490_t1/MLepR1; BEEf/BEEr; mlCOIintF/dgHCO-2198; C_LepFolF/C_LepFolR; LCOLong/C1-N-2191; LCO_Hym/C1-N-2191; LepF1/C_ANTMR1D; RonMWASPdeg_t1/LepR1; MLepF1/LepR1
Halictidae	Rophitinae		Ceblurgus	
Megachilidae	Megachilinae	Anthidiini	Anthidium	LepF1/LepR1; C_LepFolF/C_LepFolR; LCO1490_t1/HCO2198_t1; RonMWASPdeg_t1/LepR1; LepF1/C_ANTMR1D; BEEf/BeeR; MLepF1/LepR1; LCO1490/HCO2198
Megachilidae	Megachilinae	Anthidiini	Epanthidium	LepF1/LepR1
Megachilidae	Megachilinae	Anthidiini	Hypanthidioides	LepF1/LepR1; LepF1/C_ANTMR1D; RonMWASPdeg_t1/LepR1
Megachilidae	Megachilinae	Anthidiini	Hypanthidium	LepF1/LepR1; C_LepFolF/LepFolR
Megachilidae	Lithurginae	Lithurgini	Lithurgus	LepF1/LepR1; LCO1490/HCO2198; C_LepFolF/C_LepFolR; mlCOIintF/dgHCO-2198; RonMWASPdeg_t1/LepR1
Megachilidae	Lithurginae	Lithurgini	Microthurge	RonMWASPdeg_t1/LepR1
Megachilidae	Megachilinae	Megachilini	Coelioxys	LepF1/LepR1; LCO1490/HCO2198; RonMWASPdeg_t1/LepR1; C_LepFolF/C_LepFolR; BEEf/BEEr; LCO1490_t1/ HCO2198_t1; LepF1/C_ANTMR1D; LepF1/EnhLepR1; mlCOIintF/dgHCO-2198; LCOLong/C1-N-2191
Megachilidae	Megachilinae	Megachilini	Megachile	LepF1/LepR1; LCO1490/HCO2198; C_LepFolF/C_LepFolR; LCO1490_t1/HCO2198_t1; RonMWASPdeg_t1/LepR1; LepF1/C_ANTMR1D; BEEf/BEEr; LepF1/EnhLepR1; MLepF1/LepR1; LCO_Hym/C1-N-2191; MLepF1/C_LepFolR; C_VF1LFt1/C_VR1LRt1; LCO_Hym/Nancy_short; Hymeno-1/Hymeno-2; Cer_COI_F/HCO2198_t1; C_LepFolF/MLepR2; LCO1490-JJ/HCO2198-JJ

### Universal primer sets

The most widely recognised primer set for amplifying the COI (cytochrome c oxidase subunit I) region is the LCO1490 and HCO2198 primer set, also known as the Folmer primers [118]. This primer set was designed from a 710-base pair fragment of the COI region across 11 phyla and the putative phylum Vestimentifera [118]. The Folmer primers are commonly used as a starting point to amplify the COI region from any invertebrate species. However, some studies have reported issues with the Folmer primers, including low amplification success linked to high nucleotide variability in the LCO1490 primer and specimens’ age and preservation history [133, 134]. Additionally, the Folmer primers have been found to non-specifically amplify the coxA gene of the bacterial genus *Wolbachia*, which can infect various invertebrate hosts, including bees [135–137]. This off-target amplification can result in low-quality sequences and inaccurate identification [124, 125]. For example, a screening of the BOLD database reported that the highest number of unintended amplifications of *Wolbachia* DNA occurred for Hymenoptera [136]. Despite the limitations observed when using Folmer primers, many of the primers listed in Table 1 are based on them and show high similarity to nearly all bases (e.g., HCO has an A that replaces a G) [121] or are degenerate primers (e.g., dgHCO-2198) [116].

Another notable study on universal primers is by Simon et al. [121]. This study presents an extended list of primers that can be used to amplify the COI gene, among other regions. An interesting aspect of this work is the assignment of aliases to the primer names (e.g., C1-N-2191 - alias Nancy; C1-J-1751 - alias Ron). However, some authors (e.g., Gonçalves [138]; Maia et al. [139]) have used a third alias for these primers (e.g., C1-N-2191 - alias Nancy or mtD9; C1-J-1751 - alias Ron or mtD6), which can be confusing for readers. Regardless of the naming issue or the aliases used, the study provides 11 COI primers, as well as a larger number of primers for other regions that can be employed for amplifying bee species.

### The Lepidoptera primer set (and its variations)

The review highlighted the widespread use of the Lep primers [111]. Despite the primers initially being designed for the neotropical skipper butterfly *Astraptes fulgerator* (Lepidoptera) from dry museum specimens, they showed considerable amplification efficiency for a full-length COI 5’ region (~ 680 bp) in 465/484 bee samples and a shorter 350 bp product in 14 of the remaining 19 samples (in cases where LepF1 was combined with a different reverse primer than LepR1). In a separate study, a cocktail of primers [140], including the Lep and Folmer sets, was used to amplify organisms collected across Canada but had lower efficiency (< 90%) with significant variation in sequence recovery across different orders. While Diptera and Lepidoptera had the highest recovery, Coleoptera was intermediate, and Hemiptera and Hymenoptera had the lowest. Despite this variation, the Lep primers were still widely adopted for barcoding bee species, likely due to their higher amplification success compared to the C_LepFol cocktail. Table 2 shows the Lep primers that were used for almost all the bee genera that showed amplification. Many other primer sets, such as EnhLep [115], microLep [123], MLep [123], and LepF2_t1 [127], have also been developed based on the original Lep primers.

### The “Bee” primer sets

To address potential issues with the co-amplification of *Wolbachia* using the Folmer primers, Bleidorn and Henze [135] developed a new set of primers for the COI region called the BeeCox primer set. This primer set reportedly outperformed the Folmer primers, amplifying a ~ 670 bp product without co-amplifying the *Wolbachia* coxA gene. However, the BeeCox primers were tested on a limited taxon sampling of bees, so it remained unclear how they would perform across a broader range of Hymenoptera taxa.

Ramirez et al. [141] also designed a CO1 primer set specifically for the Meliponini tribe (Apidae). While this study focused on the molecular phylogeny of the stingless bee genus Melipona, the data uploaded to GenBank showed a high success rate, with the majority of amplicons being over 1000 bp in length.

Another study by Françoso and Arias [129] developed primer sets (BarbeeF, CO1-2166 F, CO1-2338 F, CO1-2248R, CO1-2386R and MtD9) to amplify the full DNA barcode (~ 620 bp) as well as mini-barcodes (175–294 bp) for corbiculate bees, including museum specimens, without evidence of *Wolbachia* amplification. However, these primers were tested on only a small subset of Apidae species and had relatively low annealing temperatures, potentially increasing the risk of non-specific results. The stronger point presented by the authors in using these primer sets was that the mini-barcodes could be overlapped and provide the complete COI barcode in case of amplification problems.

Magnacca and Brown [126] used and modified primers described by Simon et al. [121] in their study of 49 *Hylaeus* bee species (Hymenoptera: Colletidae). They employed the LCO_Hym (alias C1-J-1514) and the shortened Nancy_short (alias C1-N-2194) primers, with the latter resulting from an ordering error that, according to the authors, produced a better working version. These primers were later used for barcoding Irish solitary bees [124], whereas the LCOLong and C1-N-2663 primers were designed for more challenging taxa.

Creedy et al. [120] designed a specific primer set (BEEf/BEEr) based on 84 mitochondrial genomes from 22 genera, which amplified a ~ 418 bp sequence and was successful for up to 28 genera (please see Table 2). Villalta et al. [125] also developed the Hymeno primer set (Hymeno-1 / HCO-Hymeno-2) based on the Folmer primers [118], reporting a 70.74% successful amplification rate, although the precise length of the amplicon was not provided.

### The unpublished and hide but widely used primer sets

The review of available primers and their use for barcoding Caatinga bee species led to the identification of other primer sets that were frequently used, such as AP0 and TBCF. However, these and other commonly used primers like RonMWASPdeg_t1 and the Bterrestris set were difficult to confirm and validate. The RonMWASPdeg_t1 primer appears to be incorrectly referenced across several studies (e.g [142–145]). or even unreferenced (e.g [146, 147]. It is attributed to Smith et al. [143] in one study, Fornoff et al. [144] in another, when the only correct reference seems to be the unpublished entry in the BOLD primer database (RonMWASPdeg_t1– M. Alex Smith) [112]. According to the author, the primer was designed initially by Pfunder et al. [148], based on work by Simon et al. [121], however, available details are scarce. Similarly, the Bterrestris primer set was listed in the database under a different name (unpublished - Christian Widmann) than the published “BT” designation used in Moerman et al. [149]. This primer set was designed based on cytochrome oxidase I sequences for *Bombus terrestris*, resulting in a 290 bp product, and a related 330 bp *Bombus lucorum* primer set (BLF and BLR) was also developed. Unfortunately, mis-referencing, use of aliases, outdated records, and lack of design details were common challenges encountered for many primers in this review. For example, the AMR1deg_R primer was referenced in BOLD as belonging to Smith [113], but in that study, it was presented as C_ANTMR1D-AMR1deg_R, modified from Smith et al. [150]. While challenges exist, documenting successful DNA barcoding outcomes is essential and should be included in all relevant studies.

### The Vertebrate primer sets

Vertebrate primers designed for amphibians and fishes have also been have also been indicated in the BOLD database to barcode bees. For example, the COI-C01 and COI-C03 primers [122] intended for amphibians have been used to barcode the *Colletes* genus (Colletidae– BOLD sample ID: SNMI300), while the C_VF1LFt1 and C_VR1LRt1 primers [130] designed for fishes have been used for the *Megachile* genus (Megachilidae - BOLD sample ID: BOLD-0DKAQGB27). However, with only a single entry for each genus, there is a high risk of errors in the database, especially without associated publications that could provide more information or confirmation. Thus, when selecting primers from the BOLD database, a careful analysis is necessary to account for mismatched primer usage.

## Final remarks and future perspectives

This review presents an updated list of Caatinga biome bee species, which were last comprehensively documented by Zanella et al. [6] nearly 25 years ago. Through a thorough literature review, the study identified 75 new species and 9 new genera, though the list may still not fully reflect the biome’s bee biodiversity, as evidenced by the genus-species ratio observed for some families.

Another literature review was conducted to obtain suitable primers for the COI region to enable future barcoding of species within this biome. The study gathered  ~ 40 primer sets that, in different combinations, have been shown to work for 31 of the previously identified 86 genera. As far as the authors are aware, no other study has compiled this information, making this review an essential resource for future Caatinga bee research and barcoding efforts in other biomes. While this updated species list and primer compilation can aid future Caatinga bee research, significant work remains to be done. Barcoding bee species would allow for better characterisation of the biome and identification of distribution trends and new species/subspecies, thus expanding the bee diversity documented in this study. Additionally, this work could serve as a reliable starting point for broader efforts to increase bee barcoding and develop metabarcoding techniques. Such tools would help inform strategies to preserve bee populations and the flora dependent on their pollination.

## Electronic supplementary material

Below is the link to the electronic supplementary material.

Supplementary Material 1

Supplementary Material 2

## Data Availability

No datasets were generated or analysed during the current study.
