# Supplementary material for: Barcoding the Caatinga biome bees: a practical review
Source: Mol Biol Rep. 2025 Feb 4;52(1):196. doi: 10.1007/s11033-025-10307-7 (PMC11794421; doi:10.1007/s11033-025-10307-7)
Supplement: Supplementary file 2 — Supplementary Material 2 [file 11033_2025_10307_MOESM2_ESM.pdf]

Supplementary Data

Barcoding the Caatinga Biome Bees: A Practical Review.

Pedro Rodrigues[1#], Cláudia Teixeira[1#], Laura Guimarães[1*], Nuno G.C. Ferreira[1,2*]

[1] CIIMAR - Interdisciplinar Centre of Marine and Environmental Research, University of Porto, Terminal de Cruzeiros do Porto de Leixões, Av. General Norton de Matos, 4450-208, Matosinhos, Portugal.

[2] Cardiff University – School of Biosciences, Museum Avenue, Cardiff CF10 3AX, Wales (UK).

[#]Both authors contributed equally to the work

*Corresponding authors:

Nuno G. C. Ferreira (ngoncf@gmail.com)

Laura Guimarães (guimlid@gmail.com)

Literature review:

An extensive literature search was conducted using the SCOPUS and the Portal de Periódicos da CAPES (PC) databases for papers published between 1991 and 2024, following the preferred Reporting Items for Systematic Reviews and Meta-Analysis (PRISMA) guidelines. The query was submitted for title, abstract and keywords to retrieve records. The search inquiry included a combination of the terms “Caatinga” and “bees”. Scopus presented 159 records and PC 260 records. Two extra records were added to the analysis since they were cited in other manuscripts (Figure SD1). Retracted documents and editorial papers were not included in the data set. After duplicate removal, 279 records were then screened using the following criteria: 1) indicating a precise location within the geographical limits of the Caatinga region; 3) Records had to include the methodology by which the bee specimens were sampled and identified; 4) Records must detail the identification of the bees, at least until the species. From the analysis were excluded: a) Records for which the study was conducted in a transition area between the Caatinga and other biomes (e.g. Cerrado) and b) Records only reporting kleptoparasitic bee species. Based on the previous criteria 161 records were removed. Finally, a rigorous screening of the full text was made, remaining 85 peer-reviewed publications were included in the final dataset (Figure SD1).

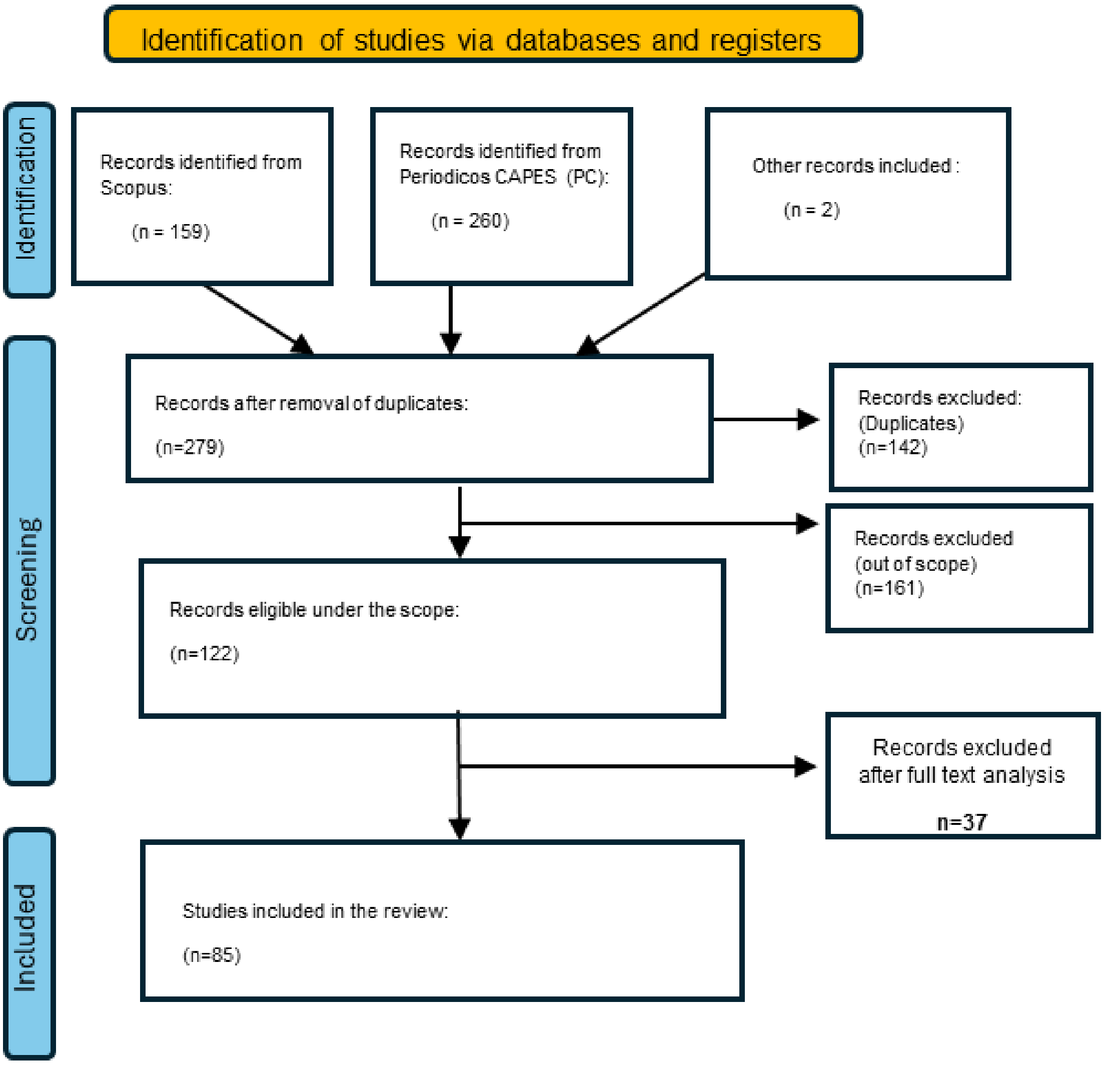

Figure SD1: PRISMA Diagram of the methodology used to include/exclude records used in this review.
